# Cluster analysis for the overall health status of elderly, multimorbid patients with diabetes

**DOI:** 10.3389/fpubh.2023.1031457

**Published:** 2023-04-04

**Authors:** Yan Bing, Lei Yuan, Ji Liu, Zezhong Wang, Lifu Chen, Jinhai Sun, Lijuan Liu

**Affiliations:** Department of Health Management, Naval Medical University, Shanghai, China

**Keywords:** multimorbidity, elderly, type 2 diabetes mellitus, overall health status, cluster group predictor

## Abstract

**Purpose:**

To evaluate the overall health status and health-related abilities and problems of elderly patients with diabetes and multimorbidity compared with those with diabetes only. Additionally, we aimed to identify different subgroups of elderly, multimorbid patients with diabetes.

**Methods:**

This cross-sectional study included 538 elderly patients with diabetes. The participants completed a series of questionnaires on self-rated health (SRH), diabetes self-management, self-efficacy, health literacy, depression, and diabetes distress. Differences in health-related abilities and problems were compared between elderly patients with diabetes and multimorbidity and those with diabetes only, with adjustments for covariates using propensity score matching. A cluster analysis was also performed to identify the overall health status subgroups of elderly, multimorbid patients with diabetes. Additionally, we conducted a multinomial logistic regression analysis to examine the predictors of health-related abilities and problem-cluster group membership.

**Results:**

Elderly patients with diabetes and multimorbidity experienced more health-related abilities and problems than those with diabetes only, particularly within the domains of depression (*p* < 0.001), and diabetes distress. The level of health literacy (*p* < 0.001) and self-management (*p* = 0.013) in elderly, multimorbid patients with diabetes was also significantly higher than that in elderly patients with diabetes only. Cluster analysis of elderly, multimorbid patients with diabetes revealed three distinct overall health status clusters. Multinomial logistic regression analysis indicated that age (OR = 1.090, *p* = 0.043), sex (OR = 0.503, *p* = 0.024), living situation (OR = 2.769, *p* = 0.011), BMI (OR = 0.838, *p* = 0.034), regular exercise (OR = 2.912, *p* = 0.041 in poor vs. good; OR = 3.510, *p* < 0.001 in intermediate vs. good), and cerebral infarction (OR = 26.280, *p* < 0.001) independently and significantly predicted cluster membership.

**Conclusion:**

Compared with elderly patients with diabetes only, those with diabetes and multimorbidity experienced more health-related abilities and problems within the domains of depression, and diabetes distress. Additionally, the level of health literacy and self-management in elderly, multimorbid patients with diabetes was significantly higher than that in those with diabetes only. Among the multimorbid diabetes group, old age, male sex, living without a partner, slightly lower BMIs, not exercising regularly, and experiencing cerebral infarctions were all positively correlated with worse overall health status.

## 1. Introduction

With aging populations and the development of improved medical care, the number of patients with chronic diseases is increasing, particularly in the elderly (1–3). According to the International Diabetes Federation (IDF) (4), the global prevalence of diabetes in 2019 was approximately 9.3% (463 million individuals). The number of adults with diabetes in China was approximately 116 million in 2019, ranking first in the world. The three main types of diabetes are type 1 diabetes mellitus (T1DM), type 2 diabetes mellitus (T2DM), and gestational diabetes mellitus (GDM), with T2DM accounting for approximately 90% of patients. The World Health Organization (WHO) defines multimorbidity as the coexistence of two or more chronic conditions in the same individual (2, 3, 5–7). As diabetes is a risk factor for many chronic diseases, such as cardiovascular and chronic kidney disease, multimorbidity is common, particularly in the elderly. In a previous study by Iglay et al. (8), a comorbidity rate of 97.5% was found in patients with T2DM (*n* = 1,389,016).

Compared with elderly patients with diabetes only, those with diabetes and multimorbidity may face more health-related problems. A meta-analysis (9) found that a reliable predictor of overall health status and death was self-rated health (SRH), and individuals with “poor” SRH had a two-fold higher mortality risk compared with those with “excellent” SRH. Additionally, Huang et al. found that people who reported more diabetes distress or depressive symptoms were more likely to have poor SRH (10). Depression, glycemic management, and complications from diabetes were all associated with SRH, and patients with diabetes who also had depression or other chronic diseases reported poorer SRH (10–12). Diabetes self-management (DSM) is a crucial behavioral element in glucose level control (13). Additionally, scientific evidence suggest that diabetes self-management is influenced by self-efficacy, depression, and diabetes distress (13–15), and is also positively correlated with health literacy (16, 17).

Self-rated health is a patient’s perception of their physical condition, and depression and diabetes distress are subjective expressions of psychological conditions. Additionally, self-management, self-efficacy, and health literacy are the patient’s perception of their disease management abilities. These six subjective indicators are connected with a patient’s perception of their health-related abilities and problems. Little emphasis has been placed on evaluating individual differences in the subjective perception of health-related abilities and problems. A general practitioner may be able to treat patients based on their individual needs. However, from a public health perspective, these subjective individual differences are also critical, as elderly, multimorbid patients with diabetes with health-related characteristics and problems associated with these domains may require different health services (18, 19). Timely treatment and demand-oriented care are vital in caring for the complex needs of patients with various health-related abilities and problems (20–22). Therefore, identifying clusters of multimorbid patients with diabetes may help to tailor continuous care programs and optimize patient-centered nursing models. Cluster analysis is useful in determining whether latent subgroups with different profiles of health-related abilities and problems exist. These analyses may also provide further examination of the factors related to overall health.

This cross-sectional study evaluated the health-related abilities, problems, and characteristics of elderly multimorbid patients with diabetes compared with elderly patients with diabetes only. Additionally, we aimed to identify different overall health status subgroups of elderly, multimorbid patients with diabetes based on self-reported, health-related abilities and problems. In doing so, we aimed to determine those with the highest need for care and support.

The current study addressed the following questions:

1. How do the health-related abilities and problems of elderly, multimorbid patients with diabetes differ from those of elderly patients with diabetes only?
2. Which subgroups of elderly, multimorbid patients with diabetes can be determined based on their self-reported health-related abilities and problems?
3. Which patient and illness-related characteristics underlie the different patterns of health-related abilities and problems among elderly, multimorbid patients with diabetes?

## 2. Methods

### 2.1. Participants and procedures

The Centers for Disease Control and Prevention in Shanghai created a health registration system for locals and later instituted a management system for patients with diabetes. Chronic disease management is within the purview of community health centers, which provide care to patients with diabetes through health registration systems for groups of patients with diabetes within neighborhoods.

We randomly selected three community health service centers from eight community health service centers in Hongkou District using SPSS software (version 26.0). From each of these centers, 200 elderly patients diagnosed with T2DM between July 2021 and October 2021were randomly selected by SPSS software and invited to participate in the cross-sectional study by their general practitioners. Patients were selected based on the following criteria: (1) diagnosis of T2DM by a certified medical practitioner, based on the 1999 WHO diagnostic criteria; (2) aged ≥65 years; (3) being non-institutionalized; (4) awareness of diagnosis; (5) having the mental capacity for participation; and (6) not being terminally ill. Patients who agreed to participate completed a series of self-report questionnaires. Logistic regression analysis was adopted in this study, and the required number of independent variables (including dummy variables) was 25 at most. According to the principle of 10 events per variable (23), the sample content was 10–20 times the number of independent variables, so the sample content was 250–500 cases. Considering that there may be invalid questionnaires caused by unavoidable factors in the investigation process, to expand the sample size and more similar to the overall population, a total of 600 questionnaires were distributed in this study. Of the 600 participants recruited, 587 completed and returned the questionnaire (97.8% response rate). Among these respondents, 538 had complete data for the variables used in cluster analyses, which we ultimately used as our sample group. The results of our comparison of the basic characteristics of the participants excluded due to missing data with those of the included group were consistent; therefore, the excluded participants had no impact on the findings of the analysis. This study was approved by the Committee on Ethics of Medicine, Naval Medical University, People’s Republic of China. Informed consent was obtained from all participants included in the study.

Prior to distributing surveys, investigators participated in standardized training on the guidelines for distributing and collecting questionnaires. Face-to-face interviews were conducted under supervision and with on-site instructions from the investigators. The investigators then confirmed the data by retrieving hemoglobin A1C (HbA1c) levels, heights, weights, treatment modalities, and diabetes complications from medical records. Quality control officers gathered the surveys on the spot and examined them for accuracy.

### 2.2. Measures

#### 2.2.1. Health literacy

We used the Health Literacy Management Scale (HeLMS) that was developed by Jordan et al. (24) and translated and culturally adapted into Chinese by Sun et al. (25, 26). This 24-item instrument assesses the ability of patients to access, understand, and use health information. The tool assesses the self-reported degree of difficulty in performing specific tasks (e.g., difficulty in reading health brochures in hospitals or clinics) using a five-point Likert scale ranging from 1 (not difficult at all) to 5 (completely impossible to do). We computed overall scores ranging from 24 to 120, with higher scores indicating a higher level of health literacy. In our sample, Cronbach’s alpha was 0.872.

#### 2.2.2. Self-efficacy

We used the Diabetes Management Self-Efficacy Scale (DMSES) that was developed by Hearnshaw and Sturt, validated by Mcdowell et al. (27), and translated and culturally adapted into Chinese by Peng et al. (28). This 20-item instrument assesses the behavioral and medical management issues related to diabetes and asks participants to report the confidence in their ability to perform specific tasks (e.g., confidence in choosing foods that are good for their health) using an 11-point Likert scale, ranging from 0 (not at all confident) to 10 (totally confident). We computed overall scores, divided them by the highest possible score on the scale, and multiplied them by 100%. A higher percentage indicated a higher perceived self-efficacy. In our sample, Cronbach’s alpha was 0.963.

#### 2.2.3. Self-management behaviors

We used the Diabetes Self-management Questionnaire (DSMQ), developed by Schmitt et al. (29) and translated and culturally adapted into Chinese by Chao-Qun Li et al. (30). This 16-item tool assesses diabetes self-care activities associated with glycemic control. Participants were asked to rate the extent to which each statement applied to their self-management activities over the previous 8 weeks. The rating scale is a four-point Likert scale ranging from 0 (does not apply to me) to 3 (applies to me very much). Certain portions of the responses were converted, and we computed overall scores ranging from 0 to 48, with higher scores indicating more effective self-care. In our sample, Cronbach’s alpha was 0.760.

#### 2.2.4. Depression

The Beck Depression Inventory (BDI) was used to analyze depression. The BDI, developed by Beck et al. (31), has 13 items and assesses depression levels throughout the previous week. Participants were asked to rate the extent to which each statement applied to their feelings on a four-point Likert scale that ranges from 0 (not at all) to 3 (totally). Overall scores ranged from 0 to 39, with higher scores indicating more depression. In our sample, Cronbach’s alpha was 0.896.

#### 2.2.5. Distress

The Diabetes Distress Scale (DDS) was used to measure levels of diabetes distress. The DDS was developed by Polonsky et al. (32) and translated and culturally adapted to Chinese by Yang et al. (33). This 17-item instrument consists of four subscales: (1) emotional burden, (2) pain related to doctors, (3) pain related to life patterns, and (4) pain related to relationships. Participants were asked to assess their degree of psychological distress using a six-point Likert scale, ranging from 0 (not a problem) to 5 (a serious problem). Although the DDS contains four subscales, each question is independent from the others; therefore, we computed average scores ranging from 0 to 5. Average scores ≥3, indicated distress from the disease. In our sample, Cronbach’s alpha was 0.959.

#### 2.2.6. Self-rated health

We assessed physiological health status using the SRH of patients, which was measured with the statement, “How do you feel about your current general health compared with your peers?” The item was scored on a five-point Likert scale (1 = very poor, 2 = poor, 3 = fair, 4 = good, and 5 = excellent), with higher scores indicating better SRH.

#### 2.2.7. Patient characteristics

Patients were asked about their age; sex (male or female); highest level of education (primary school or below, secondary school or above); living situation (with a partner, without a partner); and smoking status (currently smoking, others).

According to guidelines from the American College of Sports Medicine (34), for older adults, regular exercise includes at least 150 min a week of moderate intensity, balance training, and muscle-strengthening activities. Regular exercise status was assessed by asking participants how many days per week and how many minutes per day they exercised on average.

The heights and weights of the participants were measured by trained community health staff. Height was measured without shoes or a cap to the nearest 0.1 cm, and weight was measured to the nearest 0.1 kg. Body mass index (BMI) was calculated as the weight (kg) divided by the square of height (m).

#### 2.2.8. Illness-related characteristics

These variables included number of years with diabetes (based on the first diabetes diagnosis); family history of diabetes (yes, no); treatment type (oral antidiabetic, dietary control, and taking insulin); occurrences of hypoglycemia in recent years (yes, no); blood glucose (normal or abnormal); and glycosylated HbA1c (normal or abnormal).

The number of morbidities was measured with a 14-item list of chronic diseases commonly found among older patients with diabetes, including ketoacidosis, hyperglycemic hyperosmolar state, diabetic lactic acidosis, diabetic neuropathy, diabetic foot, kidney disease, retinopathy, hypertension, cancer, chronic gastric disease, arthritis, coronary heart disease, cerebral infarction, and chronic obstructive pulmonary disease.

Considering the different types of blood sugar collected by each community health worker, four blood glucose indicators were listed in the questionnaire, including fasting blood glucose (FBG), 2-h postprandial blood glucose, random blood glucose, and glucose tolerance test (OGTT), which were collected through both capillary blood and venous blood collection for a total of eight samples. Respondents do not need to fill in all, only need to fill in at least one of eight items.

### 2.3. Data analysis

All data collected in this study were analyzed using SPSS software (version 26.0). Data were assessed for entry errors and missing data prior to analyses. Patients with missing self-reported data on the variables of self-reported health, depression, diabetes distress, health Literacy, self-efficacy, and self-management will be excluded. Normally distributed measurement data were expressed as mean ± standard deviation (SD), whereas non-normally distributed measurement data were expressed as median (interquartile range, IQR). Comparisons of normal and non-normal data were examined using analyses of variance (ANOVAs) and Mann–Whitney tests, respectively. Categorical data were expressed as *n* (%), and the differences between the two groups were examined using chi-square analyses or Fisher’s exact tests. Statistical significance was set at *p* < 0.05.

To describe the study sample and differences between elderly multimorbid patients with diabetes and elderly patients with diabetes only, we performed a series of chi-squared tests, ANOVA, and non-parametric tests. To examine differences in health-related abilities and problems between elderly, multimorbid patients with diabetes and those with diabetes only, we performed non-parametric tests. Propensity score matching (PSM) was conducted to adjust for possible differences between the two groups in the distributions of age, sex, education level, living situation, BMI, smoking status, regular exercise, duration of diabetes, and family history of diabetes. Logistic regression was used to calculate the propensity score of each participant, and caliper matching was used to identify suitable pairs. The matching ratio was 1:1, and the caliper value was 0.05.

A two-phase clustering approach was used to investigate the patterns of overall health status among elderly, multimorbid patients with diabetes. In the first phase, R (version 4.2.2) was used to help determine the number of clusters. In the second phase, a *k*-means clustering algorithm was performed to optimize the solution, based on the findings from the first phase. We also converted the scores of the variables used for cluster analyses into standardized values prior to analysis, as each instrument had different scales and units. Differences in the variables used for cluster analysis between the subgroups were analyzed using non-parametric tests.

To examine the composition of each subgroup of elderly, multimorbid patients with diabetes and to test for differences between the subgroups regarding patient and illness-related characteristics, we performed a univariate analysis with a series of Chi-square tests and non-parametric tests. Alpha values (α) were set at 0.05.

Moreover, to analyze the predictors of health-related abilities and problem-cluster group membership, we conducted a multinomial logistic regression analysis. The cluster group number was used as the dependent variable. Statistically significant variables in univariate analyses were included in the multinomial logistic regression analysis. To assess the effectiveness of the predictors in predicting cluster group membership, ROC curve analysis was incorporated.

## 3. Results

### 3.1. Characteristics of elderly multimorbid patients with diabetes and elderly patients with diabetes only

Among the 538 patients with diabetes, 63.9% (*N* = 344) had multiple morbidities (Table 1; with further details in Supplementary Table S1) and 36.1% (*N* = 194) had only diabetes. As shown in Table 1, the “Multimorbid diabetes” group differed from the “Diabetes only” group in most aspects.

**Table 1:** Patient characteristics and illness-related characteristics of elderly, multimorbid patients with diabetes and elderly patients with diabetes only (*N* = 538).

	Multimorbid diabetes (*n* = 344)	Diabetes only (*n* = 194)	*χ^2^/H* ^*^ */F* *^#^*	*p*
*Patient characteristics*				
Age (*M*, SD)	74.49 (7.15)	73.03 (7.12)	−2.291	0.022
Gender				
Male	163 (47.4%)	81 (41.8%)	1.587	0.208
Female	181 (52.6%)	113 (58.2%)		
Education level				
Primary school or below	46 (13.4%)	36 (18.6%)	2.581	0.108
Secondary school or above	298 (86.6%)	158 (81.4%)		
Living situation				
With a partner	251 (73.0%)	166 (85.6%)	11.300	0.001
Without a partner	93 (27.0%)	28 (14.4%)		
BMI (*M*, SD)	24.31 (3.25)	23.35 (2.61)	−3.530	<0.001
Smoking status				
Currently smoking	50 (14.5%)	14 (7.2%)	6.339	0.012
Other	294 (85.5%)	180 (92.8%)		
Regular exercise				
Yes	212 (61.6%)	153 (78.9%)	16.896	<0.001
No	132 (38.4%)	41 (21.1%)		
*Illness-related characteristics*				
Duration of diabetes (Q1, Q3)	10.00 (5.00,15.00)	9.00 (5.00,14.00)	0.975	0.330
Family history of diabetes				
Yes	113 (33.3%)	45 (23.4%)	5.743	0.017
No	226 (66.7%)	147 (76.6%)		
Treatment type				
Oral antidiabetic	290 (84.3%)	154 (79.4%)	2.083	0.149
Dietary control	140 (40.7%)	60 (30.9%)	5.070	0.024
Taking insulin	57 (16.6%)	17 (8.8%)	6.373	0.012
Experienced hypoglycemia	58 (17.7%)	11 (5.9%)	14.088	<0.001
Blood glucose				
Normal	173 (50.4%)	157 (80.9%)	48.631	<0.001
Abnormal	170 (49.6%)	37 (19.1%)		
HbA1c				
Normal	151 (52.4%)	133 (80.6%)	35.605	<0.001
Abnormal	127 (47.6%)	32 (19.4%)		

* Mann–Whitney test was conducted in the duration of diabetes and the statistic is H.

# ANOVAs were conducted in age and BMI and the statistics are F.

Other variables are categorical data, which were examined by chi-square analysis and the statistics are χ^2^.

#### 3.1.1. Patient characteristics

Multimorbid patients with diabetes consisted of 163 (47.4%) men and 181 (52.6%) women aged between 65 and 94 years (M = 74.49, SD = 7.15), while patients with diabetes only consisted of 81 (41.8%) men and 113 (58.2%) women aged between 65 and 94 years (M = 73.03, SD = 7.12; *p* = 0.022). A greater number of patients with diabetes only (85.6%) lived with their partners compared with multimorbid patients with diabetes (73.0%; *p* = 0.001). The mean BMI in the multimorbid patients with diabetes group was 24.31 (SD = 3.25), which was higher than that in diabetes only group (M = 23.35, SD = 2.61; *p* < 0.001). Moreover, a greater number of multimorbid patients with diabetes (14.5%) were currently smoking compared with patients with diabetes only (7.2%; *p* = 0.012). In addition, more patients with diabetes only (78.9%) exercised regularly compared with multimorbid patients with diabetes (61.6%; *p* < 0.001). No significant differences were observed between the two groups in terms of sex or education level.

#### 3.1.2. Illness-related characteristics

More patients in the “Multimorbid diabetes” group had a family history of diabetes (*p* = 0.017), and compared with patients with diabetes only, multimorbid patients with diabetes were more likely to have abnormal HbA1c (*p* < 0.001) and blood glucose (*p* < 0.001) levels as well as have experienced hypoglycemia events (*p* < 0.001). Additionally, a greater number of multimorbid patients with diabetes were being treated with dietary control (*p* = 0.024) and insulin (*p* = 0.012) compared with the diabetes only group. No significant differences were noted between the two groups in terms of the duration of diabetes and oral antidiabetic therapy.

### 3.2. Health-related problems in elderly multimorbid patients with diabetes and elderly patients with diabetes only

Table 2 shows that, in non-parametric tests, the differences between diabetes only group and the multimorbid diabetes group were statistically significant in the domains of self-reported health (*p* = 0.047), depression (*p* < 0.001), diabetes distress (*p* < 0.001), health literacy (*p* < 0.001), and self-efficacy (*p* = 0.049). After propensity score matching, diabetes only group and the multimorbid diabetes group were not statistically significant in terms of patient and illness-related characteristics (Supplementary Table S2). Compared with those in the diabetes only group, those with multimorbidity and diabetes experienced more health-related problems within the domains of depression, and diabetes distress after adjustments by propensity score matching, while the level of health literacy (*p* < 0.001) and self-management (*p* = 0.013) in elderly, multimorbid patients with diabetes was significantly higher than that in those with diabetes only. The differences in self-efficacy and self-reported health between the two groups were no longer significant after adjustments (Supplementary Table S3).

**Table 2:** Health-related abilities and problems of elderly, multimorbid patients with diabetes versus elderly patients with diabetes only (*N* = 538).

	Score range	Median (Q1, Q3)	*Z*	*p* Crude	*p a*djusted**
		Multimorbid diabetes (*n* = 344)				Diabetes only (*n* = 194)
Self-reported health	1–5	3.00 (3.00,4.00)	3.00 (3.00,4.00)	−1.987	0.047	0.471
Depression	0–39	1.00 (0.00,3.00)	0.00 (0.00,1.00)	5.412	<0.001	<0.001
Diabetes distress	0–5	1.41 (1.06,2.04)	1.09 (1.00,1.47)	5.593	<0.001	0.001
Health literacy	24–120	65.00 (58.00,71.00)	60.00 (53.00,66.00)	5.925	<0.001	<0.001
Self-efficacy	0–100	77.00 (67.00,85.38)	81.00 (65.00,86.50)	−1.972	0.049	0.608
Self-management	0–48	33.00 (27.00,38.00)	32.00 (28.00,36.25)	0.948	0.343	0.013

** Adjusted for age, gender, education level, living situation, BMI, smoking status, regular exercise, duration of diabetes, and family history of diabetes by Propensity Score Match.

### 3.3. Patterns in the overall health status of elderly, multimorbid patients with diabetes

**Figure 1:**
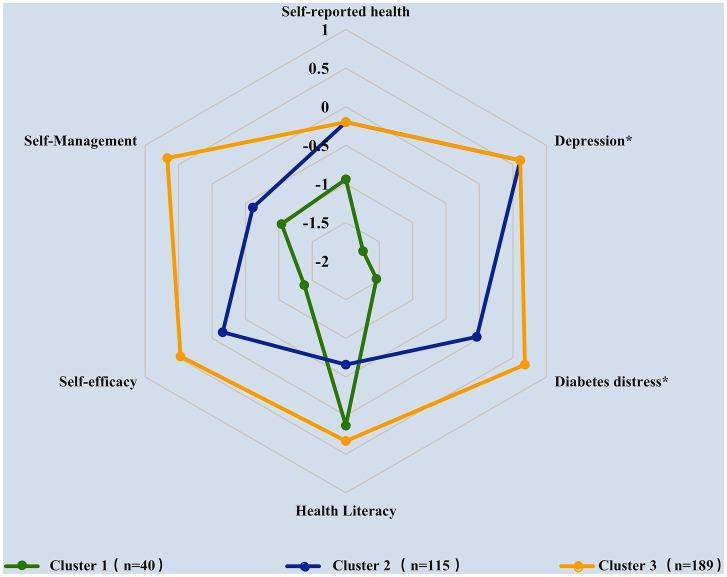
Description of elderly, multimorbid patients with diabetes clusters according to the median of standardized scores for each variable (^*^the *z*-scores of diabetes distress and depression variables were inverted).

According to the results of R (version 4.2.2), the best number of clusters is three (Supplementary Figure S1). A *k*-means iterative partitioning technique with a three-cluster solution identified three groups with distinct overall health-status profiles (Figure 1; Table 3). Figure 1 was plotted by setting each cluster’s position along the axes using the median of the z-scores of the variables used in cluster analyses. The direction of the z-score for the diabetes distress and depression variables was inverted before averaging for the consistency of higher scores representing better health status with other variables’ definitions. Therefore, the outermost graph in Figure 1 represents the best overall health status.

**Table 3:** Description of elderly, multimorbid patients with diabetes clusters according to self-reported data (*N* = 344).

	Score range	Median (Q1, Q3)	*H*	*p*
		The “poor” group (cluster 1; *n* = 40)			The “intermediate” group (cluster 2; *n* = 115)	The “good” group (cluster 3; *n* = 189)
Self-reported health	1–5	2.50 (2.00,3.00)^c^	3.00 (3.00,3.00)^b^	3.00 (3.00,4.00)^a^	82.893	<0.001
Depression	0–39	9.00 (6.00,11.75)^b^	0.00 (0.00,3.00)^a^	0.00 (0.00,2.00)^a^	99.075	<0.001
Diabetes distress	0–5	2.62 (2.18,3.16)^c^	1.65 (1.18,1.88)^b^	1.18 (1.00,1.68)^a^	98.259	<0.001
Health Literacy	24–120	66.00 (58.75,74.75)^a^	58.00 (54.00,63.00)^b^	68.00 (65.00,73.50)^a^	111.544	<0.001
Self-efficacy	0–100	49.75 (40.50,66.50)^c^	71.00 (59.50,79.50)^b^	82.00 (74.75,90.00)^a^	110.384	<0.001
Self-management	0–48	25.00 (19.00,31.75)^b^	28.00 (25.00,31.00)^b^	37.00 (33.00,40.00)^a^	164.326	<0.001

^a,b,c^ Bonferroni post-hoc tests showed that values with different superscripts differ significantly at the level of at least *p* < 0.05 (where, for example, “a” differs from “b” but not from “ab”).

Table 3 shows the median scores and IQR for each variable before standardization as well as the results of Bonferroni *post hoc* comparisons. Results indicated that the three clusters showed distinct overall patient health status patterns as well as significant differences in terms of the six variables (*p* < 0.001). According to the median of standardized scores for each variable, the overall health status of Cluster 3 was the best, followed by Cluster 2 and Cluster 1 (Figure 1), which are henceforth referred to as the “good,” “intermediate,” and “poor” groups, respectively, based on their profiles.

The poor health group, accounting for 11.6% (*n* = 40) of the multimorbid patients with diabetes, was the smallest group and was characterized by the worst relative overall health status. This cluster exhibited the highest scores in depression (*p* < 0.001) and diabetes distress (*p* < 0.001) as well as the lowest scores in self-rated health (*p* < 0.001), self-efficacy (*p* < 0.001), and self-management (*p* < 0.001).

The good health group, accounting for 54.9% (*n* = 189) of the multimorbid diabetes group, was the best in most variables, with the lowest scores in depression (*p* < 0.001) and diabetes distress (*p* < 0.001) as well as the highest scores in self-rated health (*p* < 0.001), self-efficacy (*p* < 0.001), self-management (*p* < 0.001), and health literacy (*p* < 0.001).

Finally, the intermediate health group, accounting for 33.4% (*n* = 115) of the multimorbid diabetes group, was characterized by the lowest relative scores in health literacy (*p* < 0.001).

### 3.4. Characteristics associated with the three clusters of elderly, multimorbid patients with diabetes

#### 3.4.1. Patient characteristics

As shown in Table 4 (with further details in Supplementary Table S4), patients in the poor health group were significantly older than those in the other two clusters (*p* = 0.001). The intermediate health group contained more men than women, whereas the other two clusters contained more women (*p* = 0.007). The educational level of the poor health group was lower than that in the other two clusters (*p* = 0.001). Additionally, only 45.0% of patients in the poor health group lived with a partner, compared with 69.6 and 81.0% of patients in the intermediate and good health group, respectively (*p* < 0.001). BMIs in intermediate health group were significantly higher than those in poor group (*p* = 0.044), and compared with the good health group, of which 78.3% of patients exercised regularly, only 37.5% of the patients in poor health group and 42.6% in intermediate health group maintained regular exercise (*p* < 0.001). No significant differences were noted among the three clusters in terms of smoking status.

**Table 4:** Patient and illness-related characteristics of elderly, multimorbid patients with diabetes clusters (*N* = 344).

	The “poor” group (cluster 1; *n* = 40)	The “intermediate” group (cluster 2; *n* = 115)	The “good” group (cluster 3; *n* = 189)	*χ*^2^/H^*^/F*^#^*	*p*
*Patient characteristics*					
Age (*M*, SD)	78.38 (1.40)^a^	73.98 (0.65)^b^	73.98 (0.48)^b^	6.901	0.001
Gender					
Male	13 (32.5%)^a^	67 (58.3%)^b^	83 (43.9%)^a^	9.923	0.007
Female	27 (67.5%)	48 (41.7%)	106 (56.1%)		
Education level					
Primary school or below	13 (32.5%)^a^	15 (13.0%)^b^	18 (9.5%)^b^	15.061	0.001
Secondary school or above	27 (67.5%)	100 (87.0%)	171 (90.5%)		
Living situation					
With a partner	18 (45.0%)^a^	80 (69.6%)^b^	153 (81.0%)^b^	22.645	<0.001
Without a partner	22 (55.0%)	35 (30.4%)	36 (19.0%)		
BMI (*M*, SD)	23.44 (0.50)^a^	24.84 (0.30)^b^	24.17 (0.24)^b^	3.152	0.044
Smoking status					
Currently smoking	4 (10.0%)	24 (20.9%)	22 (11.6%)	5.652	0.059
Other	36 (90.0%)	91 (79.1%)	167 (88.4%)		
Regular Exercise					
Yes	15 (37.5%)^a^	49 (42.6%)^a^	148 (78.3%)^b^	49.672	<0.001
No	25 (62.5%)	66 (57.4%)	41 (21.7%)		
*Illness-related characteristics*					
Duration of diabetes (Q1, Q3)	11.50 (7.25,21.75)^a^	7.50 (5.00,12.00)^b^	10.00 (5.00,16.00)^ab^	9.757	0.008
Family history of diabetes					
Yes	15 (37.5%)	35 (31.3%)	63 (33.7%)	0.542	0.763
No	25 (62.5%)	77 (68.8%)	124 (66.3%)		
Treatment type					
Oral antidiabetic	32 (80.0%)	92 (80.0%)	166 (87.8%)	3.946	0.139
Dietary control	15 (37.5%)	44 (38.3%)	81 (42.9%)	0.818	0.664
Taking insulin	10 (25.0%)^a^	10 (8.7%)^b^	37 (19.6%)^a^	8.45	0.015
Experienced hypoglycemia					
Yes	13 (34.2%)^a^	14 (12.8%)^b^	31 (17.2%)^ab^	8.889	0.012
No	25 (65.8%)	95 (87.2%)	149 (82.8%)		
Number of multimorbidity (Q1, Q3)	2.00 (1.00,3.00)^a^	1.00 (1.00,2.00)^b^	1.00 (1.00,2.00)^b^	17.952	<0.001
Multimorbidity					
Ketoacidosis^$^	0 (0.0%)	0 (0.0%)	1 (0.5%)		
Hyperglycemic hyperosmolar state^$^	0 (0.0%)	0 (0.0%)	0 (0.0%)		
Diabetic lactic acidosis^$^	0 (0.0%)	0 (0.0%)	0 (0.0%)		
Diabetic neuropathy	2 (5.0%)	1 (0.9%)	7 (3.7%)	2.737	0.254
Diabetic foot^$^	0 (0.0%)	0 (0.0%)	6 (3.2%)		
Kidney disease	1 (2.5%)	2 (1.7%)	6 (3.2%)	0.581	0.748
Retinopathy	2 (5.0%)	6 (5.2%)	11 (5.8%)	0.074	0.964
Hypertension	37 (92.5%)	105 (91.3%)	176 (93.1%)	0.338	0.844
Cancer	1 (2.5%)	5 (4.3%)	17 (9.0%)	3.746	0.154
Chronic gastric disease	2 (5.0%)	8 (7.0%)	7 (3.7%)	1.611	0.447
Arthritis	7 (17.5%)^a^	5 (4.3%)^b^	18 (9.5%)^ab^	6.789	0.034
Coronary heart disease	13 (32.5%)	19 (16.5%)	37 (19.6%)	4.786	0.091
Cerebral infarction	16 (40.0%)^a^	8 (7.0%)^b^	8 (4.2%)^b^	51.184	<0.001
Chronic obstructive pulmonary disease^$^	0 (0.0%)	1 (0.9%)	3 (1.6%)		
Blood glucose					
Normal	13 (32.5%)^a^	50 (43.9%)^a^	110 (58.2%)^b^	11.679	0.003
Abnormal	27 (67.5%)	64 (56.1%)	79 (41.8%)		
HbA1c					
Normal	11 (37.9%)^ab^	41 (43.6%)^b^	99 (60.0%)^a^	9.163	0.010
Abnormal	18 (62.1%)	53 (56.4%)	66 (40.0%)		

* Mann–Whitney test was conducted in the duration of diabetes and number of multimorbidity and the statistic is H.

# Analyses of variance (ANOVAs) were conducted in age and BMI and the statistics are F.

Other variables are categorical data, which were examined by chi-square analysis and the statistics are χ^2^. ^a,b^Bonferroni *post hoc* tests showed that values with different superscripts differ significantly at the level of at least *p* < 0.05(where, for example, “a” differs from “b” but not from “ab”).

$ The number of answers for some clusters was too small to calculate a reliable χ2 value for comparison.

#### 3.4.2. Illness-related characteristics

The duration of diabetes in the poor group was longer than that in the intermediate health group (*p* = 0.008). Additionally, compared with the intermediate health group, more people in poor and good health groups were on insulin therapy (*p* = 0.015). More patients in the poor health group were found to have experienced hypoglycemia compared with the intermediate group (*p* = 0.012), and the number of morbidities in the poor health group was significantly higher than that in the other two clusters (*p* < 0.001). Compared with the good group, more patients in the poor and intermediate health groups had abnormal blood glucose (*p* = 0.003) and HbA1c (*p* = 0.010) levels. Moreover, the poor health group showed a relatively high prevalence of arthritis (*p* = 0.034) and cerebral infarction (*p* < 0.001), particularly when compared with the intermediate group.

### 3.5. Multinomial logistic regression analysis of predictors in cluster group membership

As shown in Tables 5, 6 (with definitions and measurements of variables shown in Supplementary Table S5), multinomial logistic regression analyses were performed with overall health status cluster groups as a dependent variable, and with the good health group, having shown relatively high overall health status and fewer health-related problems, as the reference category. Variables with statistical significance in univariate analyses were included as covariates in the multinomial logistic regression analysis, which showed that age (OR = 1.090, *p* = 0.043), sex (OR = 0.503, *p* = 0.024), living situation (OR = 2.769, *p* = 0.011), BMI (OR = 0.838, *p* = 0.034), regular exercise (OR = 2.912, *p* = 0.041 in poor vs. good; OR = 3.510, *p* < 0.001 in intermediate vs. good), and cerebral infarction (OR = 26.280, p < 0.001) all independently and significantly predicted cluster group membership. Compared with the poor and intermediate health groups, more patients in the good health group participated in regular exercise. The good group was also younger, had higher BMIs, and had lower incidence of cerebral infarction compared with the poor health group. Additionally, the good group had more women and were more likely to live with a partner compared with the intermediate health group. To assess the effectiveness of the predictors in predicting cluster group membership, ROC curve analysis was incorporated with two individual logistic regression models: (1) poor + intermediate versus good, and (2) poor versus intermediate + good. The predictor of AUC values for “poor + intermediate versus good” is 0.836 (95% CI 0.768–0.904). The predictor of AUC values for “poor versus intermediate + good” is 0.713 (95% CI 0.657–0.770; Supplementary Figure S2).

**Table 5:** Multinomial logistic regression analysis of predictors in cluster group membership (‘poor’ vs. ‘good’).

Variable	β	Std. Error	Wald	*p*	OR	95%CI of OR	
						LCI	UCI
Intercept	−7.920	4.446	3.174	0.075			
Age*	0.086	0.043	4.097	0.043*	1.090	1.003	1.185
Gender	0.157	0.566	0.077	0.782	1.170	0.386	3.544
Education level	0.854	0.786	1.181	0.277	2.348	0.504	10.950
Living situation	1.170	0.637	3.372	0.066	3.223	0.924	11.239
BMI*	−0.177	0.083	4.518	0.034*	0.838	0.711	0.986
Regular Exercise*	1.069	0.523	4.175	0.041*	2.912	1.045	8.119
Duration of diabetes	−0.016	0.040	0.161	0.688	0.984	0.911	1.064
Taking insulin	0.826	0.624	1.752	0.186	2.285	0.672	7.766
Experienced hypoglycemia	−0.823	0.608	1.834	0.176	0.439	0.133	1.445
Number of multimorbidity	−0.279	0.273	1.051	0.305	0.756	0.443	1.290
Arthritis	0.172	1.153	0.022	0.881	1.188	0.124	11.375
Cerebral infarction*	3.269	0.888	13.540	<0.001*	26.280	4.608	149.893
HbA1c	0.698	0.522	1.787	0.181	2.010	0.722	5.596

OR, odds ratio; LCI, lower confidence interval; and UCI, upper confidence interval.

* *p* < 0.05.

**Table 6:** Multinomial logistic regression analysis of predictors in cluster group membership (“intermediate” vs. “good”).

variable	β	Std. Error	Wald	*p*	OR	95%CI of OR	
						LCI	UCI
Intercept	0.342	2.669	0.016	0.898			
Age	−0.036	0.025	1.976	0.160	0.965	0.918	1.014
Gender*	−0.686	0.305	5.078	0.024*	0.503	0.277	0.915
Education level	−0.358	0.481	0.555	0.456	0.699	0.272	1.793
Living situation*	1.018	0.401	6.458	0.011*	2.769	1.262	6.074
BMI	0.001	0.047	0.000	0.983	1.001	0.913	1.098
Regular exercise*	1.256	0.314	16.022	<0.001*	3.510	1.898	6.491
Duration of diabetes	−0.016	0.025	0.392	0.531	0.984	0.938	1.034
Taking insulin	−0.561	0.468	1.434	0.231	0.571	0.228	1.429
Experienced hypoglycemia	0.354	0.507	0.488	0.485	1.425	0.527	3.853
Number of multimorbidity	−0.119	0.156	0.581	0.446	0.888	0.653	1.206
Arthritis	−0.426	0.718	0.352	0.553	0.653	0.160	2.667
Cerebral infarction	0.185	0.694	0.071	0.789	1.204	0.309	4.692
HbA1c	0.405	0.296	1.877	0.171	1.499	0.840	2.676

OR, odds ratio; LCI, lower confidence interval; and UCI, upper confidence interval.

* *p* < 0.05.

## 4. Discussion

Considering the patient and illness-related characteristics, patients with diabetes and multimorbidity differed from those with diabetes only in almost every aspect. Moreover, compared with elderly patients with diabetes only, those with multimorbidity and diabetes experienced more health-related problems within the domains of depression and diabetes distress. Previous studies have shown that older adults with multimorbidity are at increased odds of depression in low- and middle-income countries (35), which was consistent with our findings. Although multimorbid patients with diabetes in our study were more often on dietary control and insulin therapy compared with patients with diabetes only, more patients in the multimorbid diabetes group had abnormal blood glucose and HbA1c levels as well as occurrences of hypoglycemia. An increased risk of stroke and death is associated with poor glycemic control in patients with T2DM (36). In addition, our results showed that more multimorbid patients with diabetes had a family history of diabetes compared with those with diabetes alone. Therefore, more attention to the health status of elderly multimorbid patients with diabetes is crucial.

Interestingly, the level of health literacy and self-management in elderly, multimorbid patients with diabetes was significantly higher than that in those with diabetes only. This finding was surprising as it contradicted previous reports that showed that high health literacy reduced the prevalence of multimorbidity (37, 38). Previous studies also found that patients with multiple physical and mental morbidities had outpatient visits 150% more than those with a single physical condition (39). While the literature suggests that the presence of multimorbidity may increase health literacy and self-management through regular healthcare visits and instructions from healthcare workers (40), the nature of this relationship is not well understood and may need further examination. The higher health literacy and self-management of the study subjects may be explained by the fact that the sample area is Shanghai, which is one of the most developed cities in China and has a high economic level and educational level among its residents. In addition, Shanghai community medical workers regularly organize health education classes for patients with chronic diseases, which also improves the health literacy and self-management of community residents to a certain extent.

Three distinct overall health status profiles among the elderly, multimorbid patients with diabetes in our study were identified: (1) a good overall health status group, with relatively few health-related problems across all domains; (2) a poor overall health status group; and (3) an intermediate overall health status group. The intermediate overall health status group, with comparatively lower health literacy, was characterized by more male patients, patients that did not live with a partner, and those that did not exercise regularly. Patients in this cluster may need to improve their health literacy and ability to effectively use health information and services to actively manage diabetes in their everyday lives.

The poor overall health status group had relatively more health-related problems, particularly in the SRH, depression, diabetes distress, self-management, and self-efficacy domains. These patients experienced both physical and mental health problems. These patients were also older, did not exercise regularly, had lower BMIs, and had experienced more cerebral infarctions. Based on these characteristics, this group was determined to be the most vulnerable, potentially requiring considerably more care and support.

Approximately half (55%) of the elderly, multimorbid patients with diabetes were categorized into the good health group, having experienced relatively few health-related problems. These individuals were younger, had higher levels of health literacy, and exercised more regularly. Regular exercise can help reduce the risk of cardiovascular disease, enhance cognitive function, and reduce anxiety and depressive symptoms, thus improving the mental health of patients with diabetes (41–44).

In our study, age, sex, living situation, BMI, and regular exercise were predictors of overall health status profiles, and patients in the poor overall health status group were more likely to be older. Previous studies have found that age was a significant risk factor for the prevalence of multimorbidity, which was the main risk factor of shorter life expectancy and poorer quality of life (45–47). This finding was consistent with the outcomes of the present study. Additionally, more patients in the good overall health status group lived with partners. Hopman noted that partners can provide patients with emotional support and encouragement regarding doctor visits (18). The good health group also contained more women compared with the intermediate health group. The association between sex and diabetes is a complex issue. A review (48) showed that gender differences regarding the risk, pathophysiology, and complications of T2DM varied, and that biological risk factors, psychosocial risk factors, health behavior, and pathophysiological mechanisms showed sexual dimorphism. For example, the overall higher impact of brown adipose tissue (BAT) may contribute to lower diabetes risk in women. However, women appear more sensitive to socio-contextual predictors in the development of future diabetes risk, such as education, income, and occupation.

The poor overall health group did not exercise regularly. A high level of physical activity was associated with a 35% lower risk of type 2 diabetes according to previous studies (49). Interestingly, the poor overall health group also had lower BMIs, with a mean of 23.44, compared with the intermediate and good health groups, with mean BMIs of 24.84 and 24.17, respectively. These two findings seem contradictory, as regular exercise can help shape standard body weights. However, in patients with diabetes, weight is influenced by many factors other than exercise, such as oral hypoglycemic medication. Weight loss is aided by the use of amylin mimics, metformin, sodium-glucose cotransporter 2 inhibitors, glucagon-like peptide-1 receptor agonists (GLP-1 RAs), and α-glucosidase inhibitors. Dipeptidyl peptidase-4 inhibitors and fixed-ratio insulin/GLP-1 RA combination therapy did not appear to affect body weight. Additionally, weight gain is linked to thiazolidinediones, insulin, and insulin secretagogues (50). However, specific medication information was not collected in our survey and is not discussed in this study. Furthermore, the relationship between weight and mortality risk varies in existing studies. A systematic review published in *JAMA* reported that obesity was associated with significantly higher all-cause mortality, but overweight was associated with significantly lower all-cause mortality than normal weight (51). A national cohort study from Sweden found that among older nursing home residents, obesity was associated with lower 2-year mortality (52). Further research may be required to determine the relationship between these factors.

Among the illness-related characteristics, cerebral infarction was the only variable with a significant association, emerging as an independent predictor of overall health status in our study. Previous studies have shown that a higher incidence of cerebral infarction with diabetes was associated with increased HbA1c (53), an indicator of poor glycemic control in the last 3 months. Blood glucose control was also associated with depression, diabetes distress, self-management, and self-efficacy (54, 55). Therefore, cerebral infarctions may contribute to the marked differences among the three groups.

### 4.1. Limitations

Although our study provides basic data for the management of patients with diabetes in the community, some potential limitations should be acknowledged when interpreting the results. First, this was a cross-sectional study; therefore, causal relationships could not be determined, which require longitudinal studies for validation. Second, the recruited participants were from three different community health centers in Shanghai, and some patients may not have participated in the study due to serious physical illnesses, all of which may have created selection bias. Therefore, the representativeness of the present findings for the national population may not be guaranteed. Third, although the sample size in our study was not large, it was acceptable according to the 10 events per variable principle. Additionally, the random sampling method was adopted to compensate, to some extent, for the negative impact caused by insufficient sample size. However, the representativeness of the population remains uncertain. In addition, possible unstable cluster solutions may have also been included. The deficiency of the k-means clustering algorithm is that the clustering results are easily affected by the selection of clustering centers. In the case of poor data randomization, only different local optimal solutions can be obtained. Using the default method for K-means clustering in SPSS software may also have a certain impact on the stability of clustering. Although we standardized the data and checked the outliers prior to clustering analyses, which reduced the possibility of unstable solutions to a certain extent, we could not guarantee that this was the optimal solution as our sample size was not large. Future studies with larger sample sizes and cohort study designs may be needed to confirm our findings. Cluster analyses on other patients with diabetes samples are also needed to determine whether the three clusters we identified were stable.

### 4.2. Conclusion

Overall, compared with elderly patients with diabetes only, those with diabetes and multimorbidity experienced more health-related problems within the domains of depression, and diabetes distress. The level of health literacy and self-management in elderly, multimorbid patients with diabetes was significantly higher than that in elderly patients with diabetes only. However, no differences in self-reported health or self-efficacy between the two groups were noted. Among elderly, multimorbid patients with diabetes, three distinct overall health status profiles were identified. The most vulnerable patients were older, male, and living without a partner. Additionally, this group did not exercise regularly and had had more cerebral infarctions. Overall, a strong need for care and support was observed in this group. Therefore, these characteristics may need deeper consideration when identifying target groups for comprehensive support programs and health-related care.

## Data availability statement

The raw data supporting the conclusions of this article will be made available by the authors, without undue reservation.

## Ethics statement

The studies involving human participants were reviewed and approved by approval was granted by the Committee on Ethics of Medicine, Naval Medical University, People’s Republic of China. Informed consent was obtained from all individual participants included in the study. The patients/participants provided their written informed consent to participate in this study.

## Author contributions

YB, LY, JL, ZW, LC, JS, and LL contributed to the conception and design of the study and the interpretation of the findings. JL, YB, and LY prepared the materials and collected the data. YB and ZW performed the analysis. LL, JS, and LC as the corresponding author, critically revised the manuscript and oversaw the whole project. The first draft of the manuscript was written by YB, and YB, LY, JL, ZW, LC, JS, and LL commented on the previous versions of the manuscript. All authors contributed to the article and approved the submitted version.

## Funding

This study was supported by the National Social Science Foundation of China (no. 14BGL142). The views expressed by the authors in the manuscript do not necessarily reflect those of the National Social Science Foundation of China.

## Conflict of interest

The authors declare that the research was conducted in the absence of any commercial or financial relationships that could be construed as a potential conflict of interest.

## Publisher’s note

All claims expressed in this article are solely those of the authors and do not necessarily represent those of their affiliated organizations, or those of the publisher, the editors and the reviewers. Any product that may be evaluated in this article, or claim that may be made by its manufacturer, is not guaranteed or endorsed by the publisher.

## Abbreviations

IDF: International diabetes federation
T1DM: Type 1 diabetes mellitus
T2DM: Type 2 diabetes mellitus
GDM: Gestational diabetes mellitus
WHO: World health organization
SRH: Self-rated health
DSM: Diabetes self-management
HeLMS: Health literacy management scale
DMSES: Diabetes management self-efficacy scale
DSMQ: Diabetes self-management questionnaire
BDI: Beck depression inventory
DDS: Diabetes distress scale
HbA1c: Glycosylated hemoglobin A1c
PSM: Propensity score match
BAT: Brown adipose tissue
