# Supplementary material for: Cluster analysis for the overall health status of elderly, multimorbid patients with diabetes
Source: Front Public Health. 2023 Apr 4;11:1031457. doi: 10.3389/fpubh.2023.1031457 (PMC10110949; doi:10.3389/fpubh.2023.1031457)
Supplement: Supplementary file 1 [file Data_Sheet_1.docx]

Supplementary Material

# Supplementary Tables

# Table S1 Data available of different characters

| Characters | Data available |
| --- | --- |
| Total  *Patient characteristics* | 538 |
| Age | 538 |
| Gender | 538 |
| Education level | 538 |
| Living situation | 538 |
| BMI | 538 |
| Smoking status | 538 |
| Regular Exercise | 538 |
| *Illness-related characteristics* |  |
| Duration of diabetes | 537 |
| Family history of diabetes | 531 |
| Oral antidiabetic | 538 |
| Dietary control | 538 |
| Taking insulin | 538 |
| Exercise | 538 |
| Experienced hypoglycemia | 512 |
| Blood glucose | 537 |
| HbA1c | 453 |

Table S2 Distributions of all matched variables for the two propensity score matched cohorts(*N*=368)

|  | Multimorbid diabetes(*n*=184） | Diabetes only(*n*=184） | *χ^2^/H^*^/F^#^* | *P* |
| --- | --- | --- | --- | --- |
| Age (*M*, SD) | 73.87(6.83) | 72.98(7.04) | -1.225 | 0.221 |
| Gender |  |  |  |  |
| Male | 73(39.7%) | 79(42.9%) | 0.404 | 0.597 |
| Female | 111(60.3%) | 105(57.1%) |  |  |
| Education level |  |  |  |  |
| Primary school or below | 33(17.9%) | 29(15.8%) | 0.310 | 0.676 |
| Secondary school or above | 151(82.1%) | 155(84.2%) |  |  |
| Living situation |  |  |  |  |
| With a partner | 153(83.2%) | 157(85.3%) | 0.327 | 0.668 |
| Without a partner | 31(16.8%) | 27(14.7%) |  |  |
| BMI (*M*, SD) | 23.54(3.14) | 23.42(2.61) | -0.403 | 0.687 |
| Smoking status |  |  |  |  |
| Currently smoking | 173(94.0%) | 174(94.6%) | 0.051 | 1.000 |
| Other | 11(6.0%) | 10(5.4%) |  |  |
| Regular exercise |  |  |  |  |
| Yes | 136(73.9%) | 145(78.8%) | 1.219 | 0.326 |
| No | 48(26.1%) | 39(21.2%) |  |  |
| Duration of diabetes (Q1, Q3) | 9.50(5.00,15.00) | 9.00(5.00,13.50) | 0.477 | 0.633 |
| Family history of diabetes |  |  |  |  |
| Yes | 50(27.2%) | 44(23.9%) | 0.514 | 0.550 |
| No | 134(72.8%) | 140(76.1%) |  |  |

Table S3 Health-related abilities and problems of the two propensity score matched cohorts(*N*=368)

|  | score range | Multimorbid diabetes（*n*=184） | Diabetes only（*n*=184） | *Z* | *P* |
| --- | --- | --- | --- | --- | --- |
|  |  | Median (Q1, Q3) | |  |  |
| Self-reported health | 1-5 | 3.00(3.00,4.00) | 3.00(3.00,4.00) | -0.720 | 0.471 |
| Depression | 0-39 | 0.00(0.00,3.00) | 0.00(0.00,1.00) | 3.612 | **<0.001** |
| Diabetes distress | 0-5 | 1.24(1.00,2.00) | 1.12(1.00,1.50) | 3.256 | **0.001** |
| Health Literacy | 24-120 | 66.00(60.00,71.00) | 60.00(54.00,66.00) | 5.984 | **<0.001** |
| Self-efficacy | 0-100 | 79.75(71.50,88.00) | 80.50(64.75,86.50) | 0.513 | 0.608 |
| Self-Management | 0-48 | 34.00(29.00,39.00) | 32.00(27.50,36.00) | 2.484 | **0.013** |

Table S4 Data available of different characters

| Characters | Data available |
| --- | --- |
| Total  *Patient characteristics* | 344 |
| Age | 344 |
| Gender | 344 |
| Education level | 344 |
| Living situation | 344 |
| BMI | 344 |
| Smoking status | 344 |
| Regular Exercise | 344 |
| *Illness-related characteristics* |  |
| Duration of diabetes | 343 |
| Family history of diabetes | 339 |
| Oral antidiabetic | 344 |
| Dietary control | 344 |
| Taking insulin | 344 |
| Exercise | 344 |
| Experienced hypoglycemia | 327 |
| Number of multimorbidity | 344 |
| Hypertension | 344 |
| Cancer | 344 |
| Chronic gastric disease | 344 |
| Arthritis | 344 |
| Coronary heart disease | 344 |
| Cerebral infarction | 344 |
| Transient ischemic attack | 344 |
| Blood glucose | 343 |
| HbA1c | 288 |

Table S5 Definition and measurement of variables

| Name | Assignment |
| --- | --- |
| Age | True value |
| Gender | Female=0, Male=1 |
| Education level | Primary school or below=0, Secondary school or above=1 |
| Living situation | With a partner=0, Without a partner=1 |
| BMI | True value |
| Regular Exercise | Yes=0, No=1 |
| Duration of diabetes | True value |
| Taking insulin | No=0, Yes=1 |
| Experienced hypoglycemia | Yes=0, No=1 |
| Number of multimorbidity | True value |
| Arthritis | No=0, Yes=1 |
| Cerebral infarction | No=0, Yes=1 |
| HbA1c | Normal=0, Abnormal=1 |

# Supplementary Figure


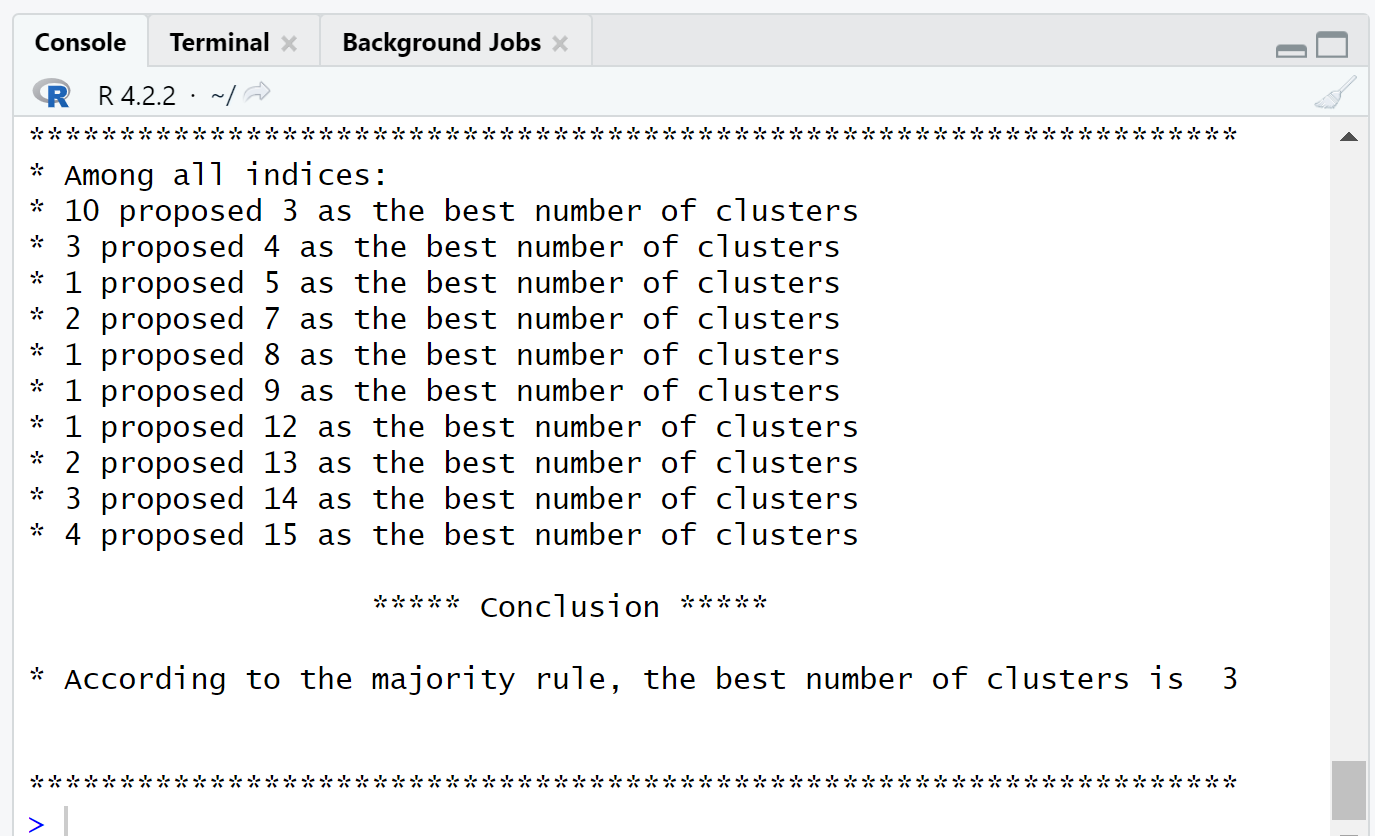


**Figure S1.** The best number of clusters according to R (version 4.2.2)


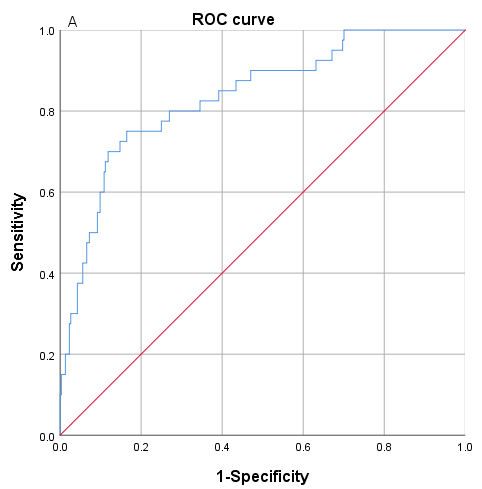

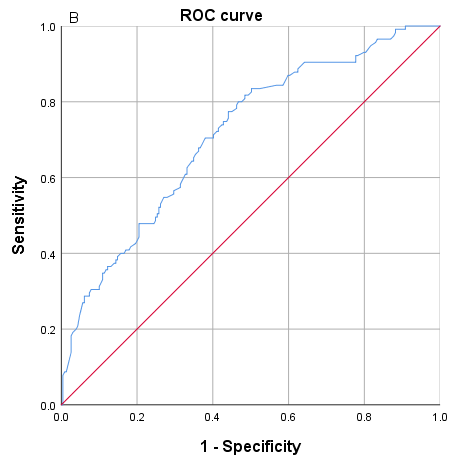


**Figure S2.** The ROC curves of the predictors in predicting cluster group membership among “poor + intermediate versus good” (A) and “poor versus intermediate + good” (B). ROC, receiver-operating characteristic; AUCs, area under curves. AUCs were 0.836 and 0.713 for predicting cluster group membership among “poor + intermediate versus good” (A) and “poor versus intermediate + good” (B), respectively.

# The R-code for the analyses was as follows:

library('NbClust')

set.seed(1234)

nb_clust <- NbClust(a1, distance = "euclidean",

min.nc=2, max.nc=15, method = "kmeans",

index = "alllong", alphaBeale = 0.1)
